# Antibiotic resistance and biofilm forming capacity of supragingival bacteria in healthy and caries patients

**DOI:** 10.3389/froh.2026.1800312

**Published:** 2026-04-24

**Authors:** David L. Auer, Moritz Pöppel, Klaus Pelz, Kirstin Vach, Cornelia Frese, Christiane von Ohle, Diana Wolff, Annette Wittmer, Fabian Cieplik, Ali Al-Ahmad

**Affiliations:** 1 Department of Operative Dentistry and Periodontology, Center for Dental Medicine, Faculty of Medicine, University of Freiburg, Freiburg, Germany; 2 Department of Medical Microbiology and Hygiene, Albert-Ludwigs-University, Freiburg, Germany; 3 Center for Data Science, Eberswalde University for Sustainable Development, Eberswalde, Germany; 4 Department of Conservative Dentistry, Clinic for Oral, Dental and Maxillofacial Diseases, University Hospital Heidelberg, Heidelberg, Germany; 5 Department of Conservative Dentistry, Periodontology and Endodontology, University Centre of Dentistry, Oral Medicine and Maxillofacial Surgery, University Hospital Tübingen, Tübingen, Germany

**Keywords:** antibiotic resistance, bacteria, caries, oral microbiology, supragingival biofilm

## Abstract

**Aim:**

The oral cavity represents a reservoir for antimicrobial resistance, and supragingival biofilms contribute to reduced antibiotic susceptibility. This study aimed to compare the antimicrobial susceptibility and biofilm-forming capacity of bacterial isolates obtained from dentally healthy individuals and participants with active carious lesions.

**Methods:**

A total of 319 bacterial isolates from 40 participants were tested for susceptibility to clinically relevant antibiotics using disk diffusion and Etest methods, supplemented by *β*-lactamase testing. Biofilm formation was quantified by crystal violet staining and categorized into three levels based on optical density. Statistical analyses accounted for clustering of isolates within individuals.

**Results:**

Resistant isolates were detected across all examined taxa. Significant group differences were observed for *Veillonella parvula* and *Lachnoanaerobaculum saburreum*. *V. parvula* isolates from caries participants showed higher proportions of intermediate or resistant classifications to ampicillin and a different distribution of biofilm categories compared to isolates from healthy individuals. For *L. saburreum*, resistant isolates from the healthy group were more frequently associated with stronger biofilm formation. Across species, stronger biofilm formation was generally associated with higher resistance among obligate anaerobes.

**Conclusion:**

Specific bacterial taxa showed distinct differences in antibiotic susceptibility and biofilm-forming capacity between caries and healthy participants. The findings indicate that supragingival biofilms from caries-active individuals may harbour altered resistance patterns, particularly among obligate anaerobic species. These isolate-level observations underscore the need to further investigate how caries-associated ecological shift in oral biofilms relate to antimicrobial resistance.

## Introduction

The human oral cavity harbors one of the most complex microbial communities in the body, which plays a decisive role in both maintaining health and promoting disease. Dental caries is among the most prevalent chronic conditions worldwide, affecting billions of people across all age groups (1–3). It develops as the result of an ecological shift within supragingival biofilms, where dietary sugars fuel acidogenic and aciduric bacteria, leading to demineralization of the dental hard tissues (4).

Oral biofilms are highly organized microbial communities embedded in a self-produced extracellular matrix, which confers protection against environmental stressors, host immune defenses, and antimicrobial agents (5–7). In this context, antimicrobial resistance (AMR) represents a major global health threat, and the oral cavity is increasingly recognized as a reservoir for resistant bacteria and transferable resistance genes (8, 9).

The biofilm lifestyle itself strongly contributes to reduced susceptibility toward antimicrobials. Restricted penetration of antimicrobial agents, altered metabolic activity, and quorum-sensing–mediated signaling all reduce bacterial susceptibility (6). Moreover, horizontal gene transfer is enhanced within biofilm communities, supporting the dissemination of resistance traits across species (10, 11). This phenomenon is particularly relevant in the supragingival environment, where numerous taxa coexist in close proximity in oral biofilms.

Recent studies have emphasized the functional consequences of caries-associated shifts in the oral microbiome showing that the addition of caries-associated bacteria to complex oral communities significantly altered biofilm composition and induced resistance- and virulence-related gene expression (12).

Few investigations, however, have systematically compared antibiotic susceptibility and biofilm-forming capacity of bacterial isolates from caries-active and caries-free subjects. Al-Ahmad et al. reported that aerobic oral species displayed stronger biofilm formation than anaerobes and that isolates with high MIC values were often moderate or strong biofilm formers (13). Yet, a direct link between caries status, resistance behavior, and biofilm-forming capacity remains largely unexplored. A persistent gap in current research concerns the extent to which caries-associated ecological shifts within supragingival biofilms are reflected in the phenotypic traits of their cultivable constituents, particularly with regard to antimicrobial resistance and biofilm formation capacity. Furthermore, comparative isolate-level studies analysing multiple species across caries statuses remain limited.

By investigating the interplay between antibiotic susceptibility, biofilm formation, and caries manifestation, this study aims to examine the association between the occurrence of caries with altered resistance patterns.

## Material and methods

### Study design and patient selection

This study involved human participants, and supragingival plaque sampling was performed under approval by the local Ethics Committees of the three Universities (604/16; S-652/2016; 863/201BO2) (Freiburg University; Heidelberg University; Tübingen University). Inclusion criteria were an age of 18–80 years in good general health and no antibiotic intake six months prior to sample collection. Exclusion criteria comprised of severe systemic diseases (e.g., diabetes mellitus, HIV), use of medications with adverse effects on the oral mucosa and pregnancy. Participants were assigned to two groups namely dentally healthy individuals (Healthy Group) and patients with active caries experience (Caries Group). In addition, data on age, sex, and regular nicotine use were obtained. Healthy individuals were defined by DMF-T index of 0, indicating dentitions free of caries and restorations. Patients were assigned to the caries group if at least two dentinal carious lesions were present at the time of sampling. The requirement of at least two active dentinal lesions was chosen to ensure that participants exhibited a clinically relevant level of ongoing cariogenic activity and to capture a supragingival biofilm environment subjected to sustained ecological pressure. Restricting the definition to a single lesion could have increased misclassification risk due to isolated or early-stage lesions and does not reliably reflect an ecologically altered cariogenic biofilm.

### Sample collection

Participants from both groups were instructed to discontinue interdental hygiene 72 h prior to sampling and to refrain from all oral hygiene measures 24 h prior to sample collection. After providing informed consent, sample collection was performed by clinical staff of the Department of Operative Dentistry and Periodontology, University Medical Center Freiburg, Department of Conservative Dentistry, Periodontology and Endodontology, University Hospital Tübingen, and the Department of Operative Dentistry, University Medical Center Heidelberg. Supragingival plaque samples were obtained from all four quadrants using a sterile Gracey curette under relative isolation with cotton rolls. The samples were collected in cryotubes (Nunc, Fisher Scientific GmbH, Schwerte, Germany) prefilled with 1.5 mL of reduced transport fluid (RTF) and immediately stored at −80 °C.

### Species isolation

Species isolation and culturing was performed according to Anderson et al. (8). Biofilm samples were thawed in a 36 °C water bath and vortexed for 30 s. Subsequently, 100 µL of the suspension was transferred into 900 µL of Peptone-Yeast medium (PY) and resuspended. 100 µL were again transferred into fresh PY medium, and the procedure was repeated three additional times. In the following, serial tenfold dilutions were prepared up to 1:10^8^. From each dilution step, 100 µL were spread onto either Columbia-Blood (CoBl) or Yeast-Cysteine-Blood (YCB) agar plates. Inoculated plates were incubated either under aerobic conditions (CoBl agar, 5%–10% CO₂) for 4–5 days or under anaerobic conditions (YCB agar, 5% CO₂, 85% N₂, 10% H₂,) in an anaerobic jar using a GENbox atmosphere generator (BioMérieux, Marcy-I Étoile, France) for 10–12 days at 37 °C, the latter allowing reliable recovery of slow-growing and fastidious obligate anaerobes, thereby ensuring adequate representation of these taxa in the culture-dependent workflow. From incubated plates, morphologically distinct colonies were isolated. Facultative anaerobic pure cultures were incubated on CoBl agar for 24 h at 37 °C. Obligate anaerobes were incubated on YCB agar for 48 h at 37 °C. After cultivation, colonies were characterized morphologically followed by matrix-assisted laser desorption/ionization time-of-flight (MALDI-TOF) species identification. Facultative anaerobic isolates were stored in cryotubes containing brain-heart infusion broth (BHI). Obligate anaerobes were stored in Basal-Glucose-Phosphate (BGP) medium with glass beads, both at −80 °C.

### Species identification and selection

For species identification, MALDI-TOF was employed. The colonies were analyzed either directly upon reading or after subculture and 24-hour incubation for aerobes and 48-hour incubation for anaerobes. For this purpose, some colony material was transferred to a target plate using a toothpick and 1 µL of 70% formic acid was added to lyse the bacterial cell walls, followed by air-drying at room temperature. 1 µL of a HCCA solution (*α*-cyano-4-hydroxycinnamic-acid) was applied to immobilize bacterial proteins in a crystalline phase and the target plate was air-dried at room temperature and introduced into a Microflex LT mass spectrometer (Bruker Daltonik GmbH, Bremen, Germany). Spectral data were analyzed using flexControl and flexAnalysis (both Bruker Daltonik GmbH, Bremen, Germany) and compared to a reference database of previously measured spectra.

### Species selection

Fifteen bacterial species (*Streptococcus oralis*, *Streptococcus anginosus*, *Streptococcus constellatus*, *Streptococcus intermedius*, *Streptococcus mutans*, *Actinomyces oris*, *Neisseria macacae*, *Neisseria mucosa*, *Capnocytophaga ochracea*, *Eikenella corrodens*, *Prevotella nigrescens*, *Fusobacterium nucleatum*, *Veillonella parvula*, *Lachnoanaerobaculum saburreum* and *Parvimonas micra*) were selected for antibiotic susceptibility testing in order to reflect the composition of the oral microbiota while including species from as many different bacterial genera as possible. *S. anginosus*, *S. constellatus*, and *S. intermedius* were collectively analyzed as the *S. anginosus* group. Similarly, *N. macacae* and *N. mucosa* were grouped together during data analysis due to their close phylogenetic relationship.

### Antimicrobial susceptibility testing

#### Antibiotic selection

Antibiotics were selected for *in vitro* testing to represent clinically relevant pharmacological classes used in dentistry, maxillofacial surgery, and general medicine. *β*-lactam antibiotics [ampicillin (AMP), cefuroxime (CXM), penicillin G (P)] were included due to their central role in the management of odontogenic infections. Macrolides [azithromycin (AZM), erythromycin (E)] and lincosamides [clindamycin (CD)] were selected as commonly chosen alternatives in patients with penicillin allergy. Fluoroquinolones [ciprofloxacin (CIP), moxifloxacin (MXF)] and tetracyclines [tetracycline (TE), tigecycline (TGC)] were included for their broad activity against oral Gram-positive and Gram-negative species. Gentamicin (CN) represented the aminoglycoside class, fosfomycin (FOS) an additional broad-spectrum agent with activity against biofilm-associated bacteria, while meropenem (MRP) and colistin (CS)were included as representative reserve antibiotics. Metronidazole (MTZ) was selected for its targeted anaerobic activity, and vancomycin (VA) as a representative glycopeptide used in severe infections. Antibiotics were selected according to the efficacy against the tested bacterial species. Facultative anaerobic species were tested against twelve antibiotics each. Obligate anaerobic species were tested with six antibiotics due to the expected large inhibition zones in the disc diffusion assay.

#### Disk diffusion assay

Cultures were screened for resistance to individual chemotherapeutic agents using the disc diffusion assay according to Kirby–Bauer (14) providing a quantitative measure of inhibition zone diameter as an indicator of sensitivity to a given antibiotic agent, excluding bacterial cultures exhibiting high susceptibility to a particular antibiotic. Previously stored pure cultures were streaked onto agar plates to obtain isolated colonies. Facultative anaerobes were incubated under aerobic conditions (5%–10% CO₂) in a CO₂ incubator on CoBl plates at 37 °C for 20–24 h. Obligate anaerobic species were incubated using a GENbox atmosphere generator (BioMérieux, Marcy-I Étoile, France) under anaerobic conditions (5% CO₂, 85% N₂, 10% H₂) on HCB plates at 37 °C for 48–72 h verified by indicator strips (Merck KGaA, Darmstadt, Germany). Morphologically similar colonies were collected from each culture, suspended in 2.5 mL of 0.9% NaCl solution and vortexed. Bacterial density of the suspension was adjusted to a McFarland standard of 0.5 for facultative anaerobes, and 0.5–1.0 was for obligate anaerobes using a turbidity meter (BioMérieux Deutschland GmbH, Nürtingen, Germany). Facultative anaerobic bacteria were tested on Mueller–Hinton agar supplemented with 5% horse blood (MHB) with the exceptions of *C. ochracea* which was tested on both MHB and Hematin agar (HA), and *E. corrodens* which was tested on CoBl agar. Testing of obligate anaerobic species was performed on Brucella blood (BB) agar. For obligate anaerobes, all suspension preparations were performed under strictly anaerobic conditions to minimize oxygen exposure and preserve viability prior to inoculation. Facultative anaerobic bacteria were evenly applied to the agar surface using a sterile swab dipped in the previously prepared NaCl solution and a plate rotator (BioMérieux Deutschland GmbH, Nürtingen, Germany). Additionally, each used swab was streaked onto a separate CoBl agar plate, which served as a control plate for subsequent Etest susceptibility testing and was incubated in a CO₂ incubator (Heraeus Deutschland GmbH & Co. KG, Hanau, Germany). Obligate anaerobic species were inoculated by pouring the entire bacterial suspension from the tube. Excess suspension was removed using a vacuum pump (Merck KGaA, Darmstadt, Germany). The plate surfaces were dried in the incubator at 37 °C for 20 min An additional YCB plate was inoculated in the same manner for subsequent Etest testing. 6 mm cellulose filter paper discs (BD Sensi-Disc™, Becton Dickinson GmbH, Heidelberg, Germany) were equilibrated to room temperature, impregnated with a single antibiotic and applied to the inoculated agar plates. Facultative anaerobes were incubated at 37 °C in a CO₂ incubator for 20–24 h, while obligate anaerobic species were incubated under anaerobic conditions at 37 °C for 48 h. The diameter of the circular inhibition zone was measured in mm and interpreted according to the recommendations of the European Committee on Antimicrobial Susceptibility Testing (15). Breakpoints were applied following a widely accepted defined hierarchy in antibiotic susceptibility testing: 1. EUCAST species-specific, 2. EUCAST genus-level or related taxa, 3. DIN 58940-3 and 4. validated literature values. Inhibition zone diameters were categorized as susceptible (s), intermediate (i), or resistant (r) based on EUCAST breakpoints (16). For species lacking established EUCAST breakpoints, reference values from the German Institute for Standardization (17), as well as publications by Gales et al. and Nagy et al., or breakpoints from species with similar expected resistance profiles were used (18, 19). Additionally, custom thresholds were defined for all antibiotics to determine when results should be validated with an Etest. These thresholds ensured that no resistant or intermediate isolate was erroneously classified as susceptible.

#### Epsilometer test

Isolates exhibiting elevated resistance were subsequently subjected to testing with Etest strips for determination minimum inhibitory concentrations (MIC) in µg/mL. Colonies were collected from the control plate, transferred into a tube containing 2.5 mL of 0.9% NaCl solution and adjusted to a McFarland turbidity as described above. The bacterial suspension was applied to the appropriate agar plates described above. An Etest strips (Liofilchem s.r.l., Roseto degli Abruzzi, Italy) was placed on each inoculated agar plate. Plates were incubated as described above and the MIC was determined at the intersection of the ellipse with the test strip. If the intersection differed between the two sides of the strip, the higher value was recorded.

#### ß-lactamase-test

For characterization of resistance profiles against *β*-lactam antibiotics and for detection of potential errors in agar diffusion susceptibility testing, Cefinase discs (Becton Dickinson GmbH, Heidelberg, Germany) were moistened with distilled water. Five isolate colonies were collected and applied to the test disc. One penicillin resistant *S. aureus* isolate was included as a positive control. After 5–60 min isolates showing a color change from yellow to red were recorded as positive, whereas no visible color change was considered negative.

#### Biofilm forming capacity assay

In order to determine the biofilm-forming capacity of each bacterial strain the biofilm formation assay was conducted as described earlier (20). In brief, an overnight culture was prepared for each strain, either in TSB for aerobic and facultative bacteria, or in GC-HP broth for obligate anaerobes. 96-well plates (Greiner Bio-One GmbH, Frickenhausen, Germany) were filled with 180 µL of TSB or GC-HP medium or 200 µL of sterile medium as negative controls. Four wells received 20 µL of a culture of *E. faecalis* as positive controls. The following four wells were inoculated with 20 µL of the bacterial cultures and incubated as described above. After 48 h, the culture medium was discarded and washed three times with 200 µL of 0.9% NaCl solution, dried at room temperature for 10 min and stained with 50 µL of 0.1% crystal violet solution. All isolates were incubated for a standardized duration of 48 h in order to ensure consistent growth conditions across all species as revealed in an earlier study by our group (13). After 10 min, the staining solution was discarded, washed three times with 200 µL of distilled water per wash and dried for 20 min at 37 °C in an incubator. 100 µL of 99% ethanol was added to each well and plates were agitated on a low-speed orbital shaker (IKA®-Werke GmbH & CO. KG, Staufen, Germany) for 20 min. The optical density (OD) of the resolubilized dye in ethanol was measured at 595 nm in a photometer (Tecan Deutschland GmbH, Crailsheim, Germany) using the i-control software (Tecan Deutschland GmbH, Crailsheim, Germany). The individual bacterial isolates were classified into three categories according to the mean OD, as described in an earlier study by our group: non-biofilm producers or C1, moderate biofilm producers or C2 or high biofilm producers or C3 (13). The low cut-off value was defined by adding 3 standard deviations of the blank to the negative control, while the high cut-off value was defined as 3 times the low cut-off value.

#### Statistical analysis

Statistical analyses were performed using STATA 16.1 (StataCorp LLC, College Station, USA). Group differences regarding antimicrobial susceptibility as well as biofilm resistance and formation were evaluated using Fisher's exact test for each individual antibiotic. To test for minimum inhibitory group differences Wilcoxon rank-sum test was used. Linear regression models were used to analyze the Inhibition Zone Diameter for each bacterium. These models provide effect size estimates with corresponding 95% confidence intervals. Additional models were run to control for influences of age and gender. The level of statistical significance was set at ɑ = 0.05. Due to the exploratory character of the study no corrections for multiple testing were made and *p*-values should therefore be interpreted cautiously.

## Results

### Patient characteristics and isolated species

Forty participants were included in the study from whom 319 bacterial isolates belonging to 15 species were isolated and tested. The median age of the participants was 26 years, ranging from 18 to 71 years. Twenty participants (50%) identified as male, twenty (50%) as female. Eight participants (20%) reported smoking at the time of sampling. The caries group comprised 17 individuals, from whom 138 bacterial isolates were obtained. The median age in this group was 32 years, ranging from 22 to 71 years. 13 participants were male (76.5%) and 4 female (23.5%). Seven participants (41.2%) in the caries group reported smoking at the time of sample collection. The healthy group comprised 23 individuals, from whom 181 bacterial isolates were obtained. The median age was 23 years, ranging from 18 to 57 years. Seven participants (30.4%) were male, 16 (69.6%) were female. One participant (0.04%) in the healthy group reported smoking at the time of sample collection.

### Antimicrobial susceptibility testing

For four (*L. saburreum*, *V. parvula*, *P. nigrescens*, *E. corrodens*, *C. ochracea*) of the twelve tested species or species groups, the proportion of resistant ratings among isolates from the caries group was higher than among those from the healthy group. However, no statistically significant group differences were observed.

### Facultative anaerobes

A total of 186 bacterial isolates were tested for susceptibility to twelve antibiotics (*S. oralis*, *S. anginosus*, *S. constellatus*, *S. intermedius*, *S. mutans*, *A. oris:* AMP, CXM, CIP, CD, E, CN, MRP, MXF, P, TE, TGC, VA; *N. macacae*, *N. mucosa*, *C. ochracea*, and *E. corrodens*: AMP, AZM, CXM, CIP, CD, CS, E, FOS, CN, MRP, TE, TGC). Detailed analysis is depicted in Table 1.

**Table 1:** Antibiotic susceptibilty testing of facultative aerobes.

Facultative Anaerobes								
Species/Group	Total isolates	Caries (*n*)	Healthy (*n*)	Overall S (%)	Overall I (%)	Overall R (%)	Caries S/I/R (%)	Healthy S/I/R (%)
*S. oralis*	39	16	23	77.4	0.9	21.8	79.2/1.0/19.8	76.1/0.7/23.2
*S. anginosus group*	24	10	14	84.7	3.5	11.8	86.7/3.3/10.0	83.3/3.6/13.1
*S. mutans*	16	15	1	84.4	5.7	9.9	84.4/5.6/10.0	83.3/8.3/8.3
*A. oris*	35	15	20	91.7	3.3	5.0	93.3/3.3/3.3	90.4/3.3/6.3
*N. macacae*/ *N. mucosa*	28	13	15	62.8	7.4	29.8	62.8/8.3/28.6	62.8/6.7/30.6
C. ochracea	22	5	17	74.2	–	25.8	75.0/–/25.0	74.0/–/26.0
E. corrodens	22	9	13	77.7	2.3	20.1	75.9/3.7/20.4	78.9/1.3/19.9

S, susceptible; I, intermediate; R, resistant. Values were calculated based on the number of isolates and rounded to one decimal place.

For *S. oralis,*16 isolates were tested. 16 isolates originated from the caries group, 23 from the healthy group. 21.8% across both participant groups were classified as resistant (R), 0.9% as intermediate (I), and 77.4% as susceptible (S)*.* (Caries group, 79.2% S, 1.0% I, 19.8% R. Healthy group 76.1% S, 0.7% I, 23.2%R).

For the *S. anginosus* group, 10 isolates of *S. anginosus*, 2 isolates of *S. constellatus* and 12 isolates of *S. intermedius* were tested. 10 isolates originated from the caries group, 14 from the healthy group 11.8% across both participant groups were classified as R, 3.5% as I, and 84.7% as S. (Caries group 86.7% S, 3.3% I and 10.0% R. Healthy group 83.3% S, 3.6% I and 13.1% R)

For *S. mutans* 16 isolates were tested. 15 isolates originated from the caries group, 1 from the healthy group. 9.9% of across both participant groups were classified as R, 5.7% as I and 84.4% as S. (Caries group 84.4% S, 5.6% I and 10.0% R. Healthy group 83.3% S, 8.3% I and 8.3% R).

For *A. oris* 35 isolates were tested. 15 isolates originated from the caries group, 20 from the healthy group. 5.0% across both participant groups were classified as R, 3.3% as I and 91.7% as S. (Caries group 93.3% S, 3.3% I and 3.3% R. Healthy group 90.4% S, 3.3% I and 6.3% R).

For *N. macacae/ N. mucosa* 28 isolates were tested. 13 isolates originated from the caries group, 15 from the healthy group. 29.8% across both participant groups were classified as R, 7.4% as I and 62.8% as S. (Caries group 62.82% S, 8.3% I and 28.6% R. Healthy group 62.8% S, 6.7% I and 30.6% R).

For *C. ochracea* 22 isolates were tested. 5 isolates originated from the caries group, 17 from the healthy group. 25.8% across both participant groups were classified as R and 74.2% as S. (Caries group 75.0% S and 25.0% R. Healthy group 74.0% S, and 26.0% R).

For *E. corrodens* 22 isolates were tested. 9 isolates originated from the caries group, 13 from the healthy group. 20.1% of across both participant groups were classified as R, 2.3% as I, and 77.7% as S. (Caries group 75.9% S, 3.7% I and 20.4% R. Healthy group 78.9% S, 1.3% I and 19.9% R).

### Obligate anaerobes

A total of 133 isolates (*P. nigrescens*, *F. nucleatum*, *L. saburreum*, *P. micra, V. parvula*) were tested for susceptibility to six antibiotics (AMP, CD, MTZ, MXF, P, TE). Detailed analysis is depicted in Table 2. Across all obligate anaerobic species, a significant difference between the healthy and the caries group could be examined for *V. parvula* and the antibiotic Ampicillin.

**Table 2:** Antibiotic susceptibilty testing of obligate aerobes.

Obligate Anaerobes								
Species/Group	Total isolates	Caries (*n*)	Healthy (*n*)	Overall S (%)	Overall I (%)	Overall R (%)	Caries S/I/R (%)	Healthy S/I/R (%)
*P. nigrescens*	24	14	10	86.1	0.7	13.2	91.7/–/8.3	78.3/1.7/20.0
*F. nucleatum*	37	12	25	91.9	–	8.1	95.8/–/4.1	80.0/–/10.0
*L. saburreum*	26	12	14	70.5	–	29.5	69.4/–/30.6	71.4/–/28.6
*P. micra*	10	3	7	96.7	–	3.3	100.0/–/–	95.2/–/4.8
*V. parvula*	36	14	22	61.6	9.7	28.7	50.0/17.9/32.1	68.9/4.6/26.5

S, susceptible; I, intermediate; R, resistant. Values were calculated based on the number of isolates and rounded to one decimal place.

For *P. nigrescens* 24 isolates were tested.14 isolates originated from the caries group, 10 from the healthy group. 13.2% across both participant groups were classified as R, 0.7% as I, and 86.1% as S. (Caries group 91.7% S and 8.3% R. Healthy group 78.3% S, 1.7% I and 20.0% R).

For *F. nucleatum* 37 isolates were tested.12 isolates originated from the caries group, 25 from the healthy group. 8.1% across both participant groups were classified as R and 91.9% as S. (Caries group 95.8% S and 4.1% R. Healthy group 80.0% S, and 10.0% R).

**Figure 1:**
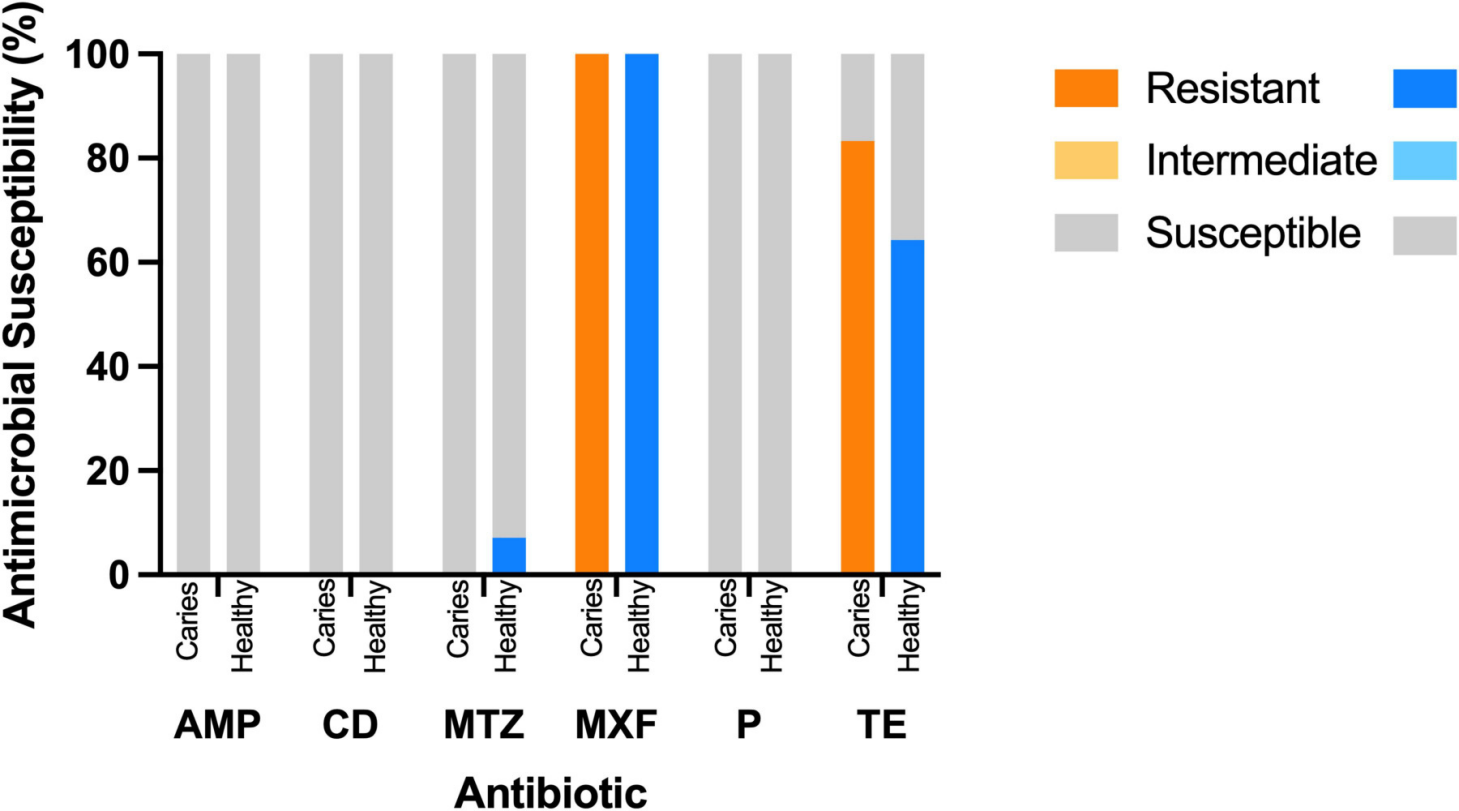
Antibiotic susceptibility testing of *L. saburreum*. AMP, ampicillin; CD, clindamycin; MTZ, metronidazol; MXF, moxifloxacin; P, penicillin G; TE, tetracyclin.

For *L. saburreum* 26 isolates were tested. 12 isolates originated from the caries group, 14 from the healthy group. Detailed analysis is depicted in Table 3. 29.5% across both participant groups were classified as R and 70.5% as S. (Caries group 69.4% S and 30.6% R. Healthy group 71.4% S and 28.6% R). The results of antibiotic susceptibility testing for *L. saburreum* are depicted in Figure 1.

**Table 3:** Antibiotic susceptibilty testing of *L. saburreum* isolates.

Antibiotic	Inhibition Zone Diameter (mm)				Minimum Inhibitory Concentration (µg/mL)				Antimicrobial Susceptibility (%)		*p*-value
	Min - Max		Mean ± SD		Min - Max		Mean ± SD		S/I/R		
	caries group	healthy group	caries group	healthy group	caries group	healthy group	caries group	healthy group	caries group	healthy group	
Ampicillin	31–44	25–45	35.4 ± 3.7	34.9 ± 5.6		0.5- 0.5		0.5±*	100.0/-/-	100.0/-/-	n.a.
Clindamycin	29–34	23–38	31.5 ± 2.1	28.9 ± 4.0	0.1–0.1		0.1±*		100.0/-/-	100.0/-/-	n.a.
Metronidazol	25–35	13–35	30.3 ± 3.2	28.6 ± 5.3	0.5–0.5	0.75–6	0.5±*	2.6 ± 3.0	100.0/-/-	92.9/-/7.1	1.00
Moxifloxacin	6–12	6–12	8.5 ± 2.4	8.4 ± 2.0	6 - >32	8 - >32	*	*	-/-/100.0	-/-/100.0	n.a.
Penicillin G	27–38	28–42	33.7 ± 2.9	34.4 ± 4.8	0.1–0.1		0.1 ± 0.0		100.0/-/-	100.0/-/-	n.a.
Tetracyclin	11–36	12–42	16.8 ± 8.8	21.9 ± 11.6	6–16	4–12	8.8 ± 3.9	7.1 ± 2.5	16.7/-/83.3	35.7/-/64.3	0.391
Total									69.4/-/30.6	71.4/-/28.6	
Overall Total									70.5/-/29.5		

S, susceptible; I, intermediate; R, resistant.

*calculation not possible; n.a.: statistical comparison not applicable because susceptibility patterns were identical in both groups. *p*-values were calculated using Fisher's exact test. Values were calculated based on the number of isolates and rounded to one decimal place.

For *P. micra* 10 isolates were tested. 3 isolates originated from the caries group, 7 from the healthy group. 3.3% across both participant groups were classified as R, and 96.7% as S. (Caries group 100.0% S. Healthy group 95.2% S and 4.8% R).

**Figure 2:**
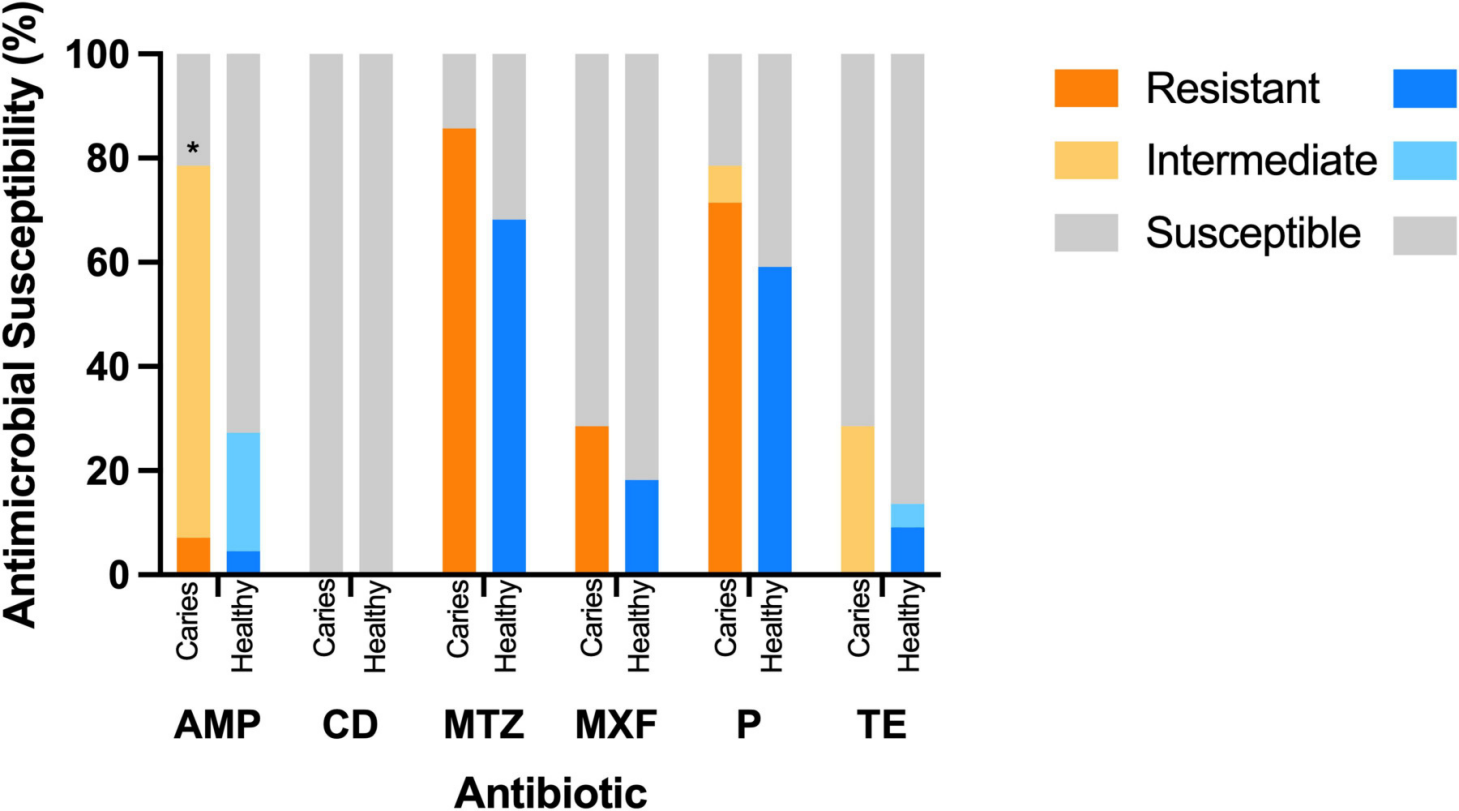
Antibiotic susceptibility testing of *V. parvula*. AMP, ampicillin; CD, clindamycin; MTZ, metronidazol; MXF, moxifloxacin; P, penicillin G; TE, tetracyclin. Asteriks indicate significant results.

For *V. parvula* 36 isolates were tested. Fourteen isolates originated from the caries group, 22 from the healthy group. Detailed analysis is depicted in Table 4. 28.7% across both participant groups were classified as R, 9.7% as I and 61.6% as S. In the caries group, 50.0% were classified as S, 17.9% as I and 32.1% as R. For Ampicillin, 21.4% were classified as S, 71.4% as I and 7.1% as R. In the healthy group, 68.9% were classified as S, 4.6% as I and 26.5% as R. For Ampicillin 72.7% were classified as S, 22.7% as I and 4.6% as R. Accordingly, a greater number of test results in the caries group were classified as resistant compared to the healthy group. Metronidazole showed the highest proportion of resistant results, with 85.7% in caries patients and 68.2% in healthy participants. Statistical analysis revealed a significant difference between the healthy and caries group for Ampicillin regarding the high number of intermediate ratings in the caries group (*p* = 0.006). The results of antibiotic susceptibility testing for V. parvula are depicted in Figure 2.

**Table 4:** Antibiotic susceptibilty testing of *V. parvula* isolates.

Antibiotic	Inhibition Zone Diameter (mm)				Minimum Inhibitory Concentration (µg/mL)				Antimicrobial Susceptibility (%)		*p*-value
	Min - Max		Mean ± SD		Min - Max		Mean ± SD		S/I/R		
	caries group	healthy group	caries group	healthy group	caries group	healthy group	caries group	healthy group	caries group	healthy group	
Ampicillin	11–23	6–27	17.8 ± 3.6	18.8 ± 6.0	0.5–12	<0,016–4	2.6 ± 4.6	*	21.4/71.4/7.1	72.7/22.7/4.6	0.006
Clindamycin	15–22	16–24	18.9 ± 2.1	20.1 ± 2.2	0.2–1	0.1–0.4	0.3 ± 0.2	0.3 ± 0.1	100.0/-/-	100.0/-/-	n.a.
Metronidazol	6–19	6–18	11.3 ± 3.4	10.4 ± 4.2	4–24	6 - >256	10.9 ± 5.5	*	14.3/-/85.7	31.8/-/68.2	0.432
Moxifloxacin	13–24	15–24	18.1 ± 2.9	20.6 ± 2.8	0.1–0.5	0.1–0.8	0.3 ± 0.2	0.4 ± 0.2	71.4/-/28.6	81.8/-/18.2	0.683
Penicillin G	9–23	6–27	17.2 ± 4.3	18.7 ± 6.0	0.4–12	0.3–32	2.9 ± 3.2	6.1 ± 11.2	21.4/7.1/71.4	40.9/-/59.1	0.274
Tetracyclin	17–25	14–27	21.8 ± 2.7	23.1 ± 3.4	0.8–0.8	12–24	0.8 ± 0.00	18.0 ± 8.5	71.4/28.6/-	86.4/4.6/9.1	0.124
Total									50.0/17.9/32.1	68.9/4.6/26.5	
Overall Total									61.6/9.7/28.7		

S, susceptible; I, intermediate; R, resistant.

*calculation not possible*.* n.a.: statistical comparison not applicable because susceptibility patterns were identical in both groups. *p*-values were calculated using Fisher's exact test. Values were calculated based on the number of isolates and rounded to one decimal place.

Comparing inhibition zone diameters across the healthy and the caries groups, a borderline significance was observed for *L. saburreum* and clindamycin in the unadjusted model (*p* = 0.049), with larger inhibition zones in the healthy group. However, this difference was no longer statistically significant after adjustment for age, sex, and smoking status.

For *V. parvula* and moxifloxacin, significantly smaller inhibition zones in isolates from healthy subjects compared with the caries group were observed (*p* = 0.019), remaining significant after adjustment for age, sex, and smoking status (*p* = 0.038).

### ß-lactamase-test

The presence of beta-lactamases was confirmed in eight bacterial isolates. The resistance of these isolates to penicillin and amoxicillin is attributable to the activity of beta-lactamases. 2.5% of the 319 bacterial cultures tested positive. Seven positive results were observed in healthy participants, and one in a caries patient. The majority of positive results occurred in *F. nucleatum*, with 16.2% of the cultures testing positive. In *P. nigrescens*, 8.3% cultures tested positive. Due to the low number of positive results for *P. nigrescens*, further statistical analysis was not performed. For *F. nucleatum*, a group comparison was not possible, as all positive results were from the healthy participant group.

### Biofilm forming capacity

One hundred eighty-six bacterial isolates (Caries group: 83, Healthy group: 103) from facultative anaerobic species and 133 bacterial isolates (Caries group: 55, Healthy group: 78) from obligate anaerobic species were tested. The highest proportion of resistant isolates was found in biofilm formation category C2. Taking into account oxygen metabolism, resistant isolates in facultative anaerobic species were more frequently associated with C3. Among obligate anaerobic bacteria, the majority of resistant results were observed in C1. Obligate anaerobic bacterial species showed considerably fewer C2 and C3 results compared to facultative anaerobic species.

Of all 319 bacterial isolates tested, 30.7% were classified as C1, 38.2% as C2, and 31.0% as C3. Among the 138 caries group isolates, 30.4% were classified as C1, 42.0% as C2, and 27.5% as C3. Among the 181 healthy group 30.9% were classified as C1, 35.4% as C2 and 33.7% as C3. Thus, a higher proportion of isolates from the healthy group contributed to C3 compared to the caries group. Regardless of the patient group, 3.8% of obligate anaerobic isolates contributed to C3 compared to 50.5% of facultative anaerobic isolates. C2 classifications accounted for 24.8% of obligate anaerobic species, compared to 47.9% among facultative anaerobic species. C1 classifications represented 71.4% of obligate anaerobic species and 1.6% of facultative anaerobic species. Accordingly, facultative anaerobic species displayed stronger biofilm formation capacity than obligate anaerobic species. When considering all test results irrespective of bacterial species, no significant differences in biofilm formation were observed between the caries and healthy groups (*p* = 0.524)

**Figure 3:**
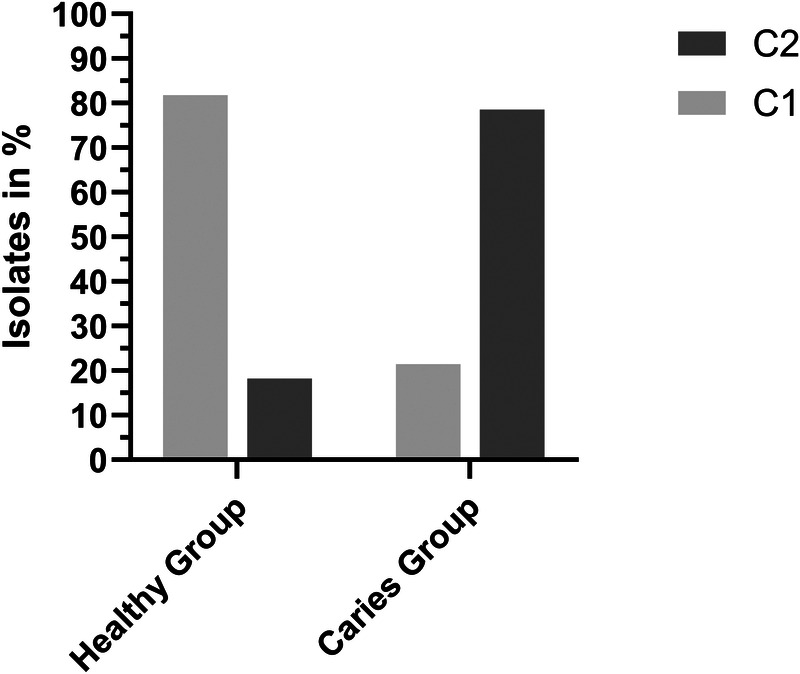
Biofilm forming capacity of *V. parvula*. AMP, ampicillin; CD, clindamycin; MTZ, metronidazol; MXF, moxifloxacin; P, penicillin G; TE, tetracyclin.

For *V. parvula*, results of the biofilm forming capacity assay are depicted in Figure 3. Of 36 Isolates tested, 14 were isolated from the caries group and 22 from the healthy group. Of the Isolates from the caries group, 21.43% (*n* = 3) were classified as C1 and 78.57% (*n* = 11) as C2. Of the Isolates from the healthy group, 81.82% (*n* = 18) were classified as C1 and 18.18% (*n* = 4) as C2. Statistical Analysis revealed a significant difference between the healthy and the caries group (*p* < 0.001). Detailed analysis is depicted in the Supplementary Table S1.

**Figure 4:**
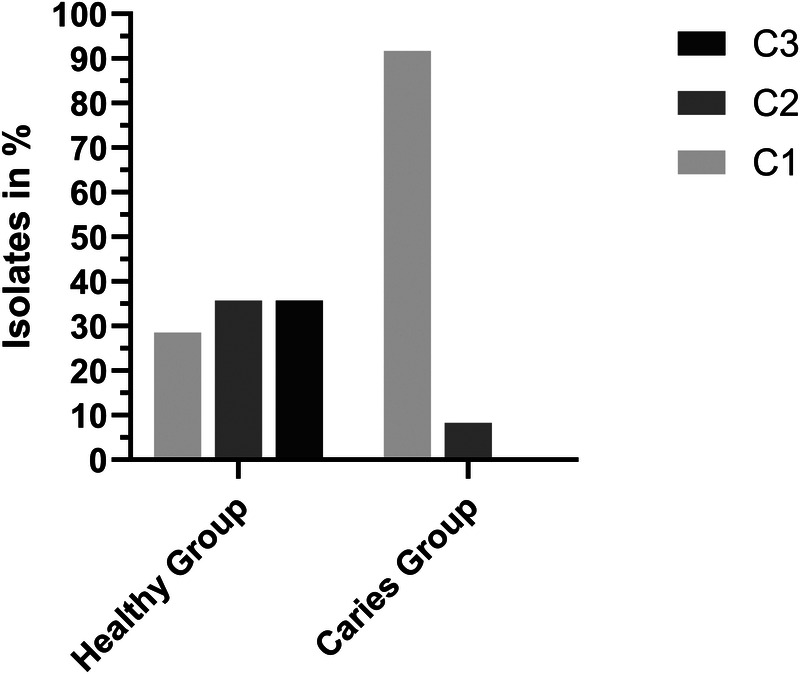
Biofilm forming capacity of *L. saburreum*. AMP, ampicillin; CD, clindamycin; MTZ, metronidazol; MXF, moxifloxacin; P, penicillin G; TE, tetracyclin.

For *L. saburreum*, results of the biofilm forming capacity assay are depicted in Figure 4. Of the 26 Isolates tested, 12 were isolated from the caries group and 14 from the healthy group. Of the Isolates from the caries group, 91.67% (*n* = 11) were classified as C1 and 8.33% (*n* = 1) as C2. Of the Isolates from the healthy group, 28.57% (*n* = 4) were classified as C1, 35.71% (*n* = 5) as C2 and 35.71% (*n* = 5) as C3. Statistical Analysis revealed a significant difference between the healthy and the caries group (*p* = 0.004). Detailed analysis is depicted in the Supplementary Table S2.

### Biofilm formation and antibiotic resistance

When evaluating the association between antibiotic susceptibility and biofilm-forming capacity independently of the caries and healthy groups, significant differences were detected between the biofilm categories in Gram-negative facultative anaerobic species. In biofilm category C3, a higher proportion of intermediate ratings (5.5%) was recorded compared to C1 (2.8%) and C2 (2.0%). However, the highest proportion of resistant results was observed in category C1 (30.6%) compared to C2 (23.9%) and C3 (27.1%). For obligate anaerobic species, the proportion of resistant results among strong biofilm formers was significantly higher than in non-biofilm formers. In biofilm category C3 a higher proportion of resistant ratings (30.0%) was recorded compared to C1 (15.6%) and C2 (24.8%).

For *V. parvula* a significance value of p_MTZ_=0.007 for metronidazole indicated a significant difference between the caries and healthy group. In the caries group, 75.0% of resistant results for MTZ were found in C2, whereas in the healthy group 80.0% were found in C1. For penicillin G with a significance value of p*P* = 0.04, 70.0% of resistant results in the caries group were found in C2, whereas in the healthy group 76.9% were observed in C1. Accordingly, isolates from the caries group exhibited significantly more resistance to metronidazole in category C2 than isolates from healthy subjects.

For *L. saburreum* resistance values in the healthy group for moxifloxacin (p_MXF_=0.004) and for tetracycline in the caries group (p_TE_=0.031) were noteworthy. In the healthy group, resistant results for MXF were distributed across all three biofilm categories (C1: 28.6%, C2: 35.7%, C3: 35.7%). In contrast, in the caries group 91.7% of resistant results were recorded in C1, 8.3% in C2, and none in C3. For TE, resistant results in the healthy group were again evenly distributed (C1: 33.3%, C2: 33.3%, C3: 33.3%). Among isolates from the caries group, however, 90.0% of resistant results were found in C1, 10.0% in C2, and none in C3. Thus, in the healthy group, resistant results were significantly more frequently associated with stronger biofilm formation.

## Discussion

The aim of the present study was to asses antimicrobial susceptibility and biofilm forming capacity of oral bacterial species isolated from participants with active carious lesions and healthy participants *in vitro*. Antimicrobial susceptibility testing revealed resistant isolates in all bacterial species. For most taxa, intergroup differences in antimicrobial resistance were modest and statistically non-significant, indicating that resistance patterns may be driven more by species-specific characteristics than by caries status alone. For the species *V. parvula* however, these findings were significantly different between the healthy and caries groups with respect to the distribution of the categories susceptible, intermediate, and resistant for ampicillin. In the caries group, significantly more isolates of *V. parvula* were classified as intermediate, whereas the majority of isolates in the healthy group were categorized as susceptible. Resistance was observed for all antibiotics (ampicillin, metronidazole, moxifloxacin, penicillin, and tetracycline) except clindamycin, indicating higher resistance rates and thus an altered resistance profile, in the caries group. This may be attributable to the close relationship between *V. parvula* and *S. mutans*. Accordingly, in a study by Schwarz et al. it could be shown, that when mimicking caries conditions in saliva-derived microcosm biofilms *S. mutans* and *V. parvula* strongly dominated microbial communities (21). While it is known that *V. parvula* metabolizes lactic acid produced by *S. mutans* (22) the precise interactions between these species remain poorly understood. Kara et al. found altered metabolic properties when both species coexisted in a dual-species biofilms due to reduced lactic acid accumulation after 48 and 72 h (23). Luppens et al. further described changes in the physiology of *S. mutans* when grown in association with *V. parvula* in a dual-species biofilm (24). In contrast to both above mentioned studies, the present study focused on clinical isolates and phenotypic susceptibility testing rather than experimentally constructed biofilm communities. Given that *S. mutans* plays an important role in the development of dental caries, these findings support the hypothesis that an increased prevalence of caries may be accompanied by altered properties of *V. parvula*. In this study, in *V. parvula*, moderate biofilm formers predominated significantly in the caries group, whereas non-biofilm formers were more prevalent in the healthy group. The distribution of isolates across the C1–C3 biofilm categories varied substantially between species and between the study groups, reflecting species-specific differences in biofilm-forming capacity that limit direct comparability across taxa.

The analysis of inhibition zone diameters revealed largely comparable susceptibility patterns between isolates from healthy and caries groups. In contrast, isolates of *V. parvula* exhibited significantly smaller inhibition zones for moxifloxacin in the healthy group, also after adjustment for potential confounders. However, given the exploratory nature of the present pilot study and the number of antibiotics tested, these findings should be interpreted with caution. Only a limited number of statistically significant differences were detected, and the observed associations require confirmation in larger studies incorporating broader microbial sampling and more advanced statistical modelling approaches. A further limitation is that antimicrobial susceptibility testing was performed at the level of individual bacterial isolates, and therefore analyses were conducted at the isolate level. Because multiple isolates may originate from the same participant, a certain degree of intra-subject dependency cannot be excluded. However, the relatively small number of participants and isolates per species limited the feasibility of more complex multilevel modelling approaches. Consequently, the findings should be interpreted as exploratory and hypothesis-generating.

Bhatti and Frank identified penicillin as the drug of choice for infections with *Veillonella* spp (25). However, in the present study, isolates were identified that displayed only intermediate susceptibility or resistance to ampicillin and penicillin. Maestre et al. did not detect resistant or intermediate isolates to clindamycin and resistance to metronidazole which is consistent with the findings of the present study although comparison remains difficult, as the authors applied different breakpoints (26). A comparison of antimicrobial susceptibility and biofilm-forming capacity between caries patients and healthy individuals has thus far rarely been described. Kouidhi et al. investigated the antibiotic susceptibility and biofilm formation of oral members of the genus *Enterococcus* from caries-active children. While attributing an important role in enterococcal infections to the high resistance rates and strong biofilm formation, they did not establish a direct link between antimicrobial susceptibility and enhanced biofilm formation (27). In a previous study, our group examined bacterial isolates from root canals of endodontically treated teeth with respect to their susceptibility to eleven antibiotics and their biofilm-forming capacity (13). There it was demonstrated that elevated MIC values can be associated with increased biofilm formation, suggesting enhanced horizontal gene transfer within biofilms as a possible explanation for the dissemination of resistance genes, assuming that the occurrence of caries reflects a longer maturation process of the supragingival biofilm before mechanical cleaning takes place. Although gene transfer is known to be enhanced in such biofilms (10, 11), a significant difference between caries patients and healthy individuals could only be demonstrated for *V. parvula*. In this case, increased biofilm formation was associated with higher resistance. As this study was based solely on culture technique, only culturable bacteria could be analyzed and no ecological interactions within multispecies biofilms could be inferred, potentially limiting generalizability. Due to the strong dependence of supragingival biofilm development on dietary habits and oral hygiene practices the duration of restricted oral hygiene in the study design may have been too short to allow a caries-associated microbiota for the exchange of resistance genes in detectable quantities in other species. A further limitation concerns the definition of the healthy group using a DMF-T score of 0. While this criterion ensures the absence of both current and past caries experience, it represents a strict requirement that may reduce the representativeness of the healthy cohort, particularly in older adults. In addition, the multiple thawing and transfer steps inherent to the isolation protocol may have selectively reduced the viability of strict anaerobes, potentially influencing the species distribution recovered for analysis. Furthermore, some species were represented by very few isolates in one of the study groups, which restricts the robustness of intergroup comparisons and reduces statistical interpretability for these taxa. Findings for species with extremely small sample sizes, such as *S. mutans* in the healthy group, should therefore be interpreted with caution. For species represented by very few isolates, such as *Parvimonas micra*, statistical inference is inherently limited. Findings for these sparsely represented taxa should therefore be regarded as descriptive, and conclusions about differences between the healthy and caries groups must be interpreted with caution.

Antimicrobial susceptibility testing was performed at the level of individual bacterial isolates, and therefore analyses were conducted at the isolate level. Because multiple isolates may originate from the same participant, a certain degree of intra-subject dependency cannot be excluded. However, the relatively small number of participants and isolates per species limited the feasibility of more complex multilevel modelling approaches. Consequently, the findings should be interpreted as exploratory and hypothesis-generating.

Conversely, due to the high diversity of supragingival biofilms in both groups, similar results for the antibiotic resistance of bacterial isolates from both groups could be anticipated. A previous comprehensive study using a metagenomic approach showed that oral biofilms are reservoirs of antibiotic resistance genes, even in individuals free from caries (8). However, the aforementioned study did not investigate the correlation between biofilm formation capacity and antibiotic resistance.

In isolates of *L. saburreum*, resistance to metronidazole, moxifloxacin, and tetracycline was detected. *L. saburreum*, formerly known as *Eubacterium saburreum*, is an acid producing, Gram-positive spore-forming rod originally isolated from tongue plaque (Saburra lingualis) (28). Resistance to metronidazole occurred exclusively in the healthy group, whereas tetracycline resistance was more prevalent in the caries group. Feres et al. also reported the presence of *L. saburreum* in the oral cavity following antimicrobial therapy with metronidazole, indicating resistance to this antibiotic (29). Significant differences between the caries and healthy groups were also observed for *L. saburreum*. In the healthy group, resistance was significantly more often associated with stronger biofilm formation, comprising of non-biofilm formers, moderate biofilm formers and strong biofilm formers, whereas in the caries group, no strong biofilm formers were identified, and non-biofilm formers predominated. A study by Mashimo et al. suggested a symbiotic relationship between *V. parvula* and *L. saburreum* (30). The authors reported altered metabolic characteristics of *V. parvula* and an increased acid content of the biofilm when cultured in association with *L. saburreum*. Whether this interspecies interaction is causally related to the observed group differences remains to be clarified in future research.

In *V. parvula*, isolates from the caries group exhibited significantly higher resistance to metronidazole and penicillin G among stronger biofilm formers compared to the healthy group. In contrast, in *L. saburreum*, resistance in the healthy group was significantly more frequently associated with stronger biofilm formation. This seemingly counterintuitive finding, is however consistent with the ecology of early supragingival biofilms. Supragingival plaque on clinically sound surfaces is typically dominated by *non-mutans acidogenic but less aciduric taxa*, including non-mutans streptococci and *Actinomyces or Schaalia* spp*.***,** which are metabolically adapted to the mildly acidic and nutritionally fluctuating conditions characteristic of health-associated biofilms. These taxa can lower pH to levels sufficient to demineralize enamel despite not being as aciduric as mutans streptococci or lactobacilli, and they possess diverse metabolic capabilities - including glycoprotein degradation and alkali production - that promote persistence and biofilm stability under healthy conditions (31)*.* Furthermore, metagenomic analyses of supragingival plaque have demonstrated that health-associated communities frequently show higher taxonomic diversity**,** with enrichment of taxa capable of flexible metabolic strategies and stable co-occurrence patterns within the early biofilm matrix (32)*.* These ecological features favor robust biofilm formation even in the absence of caries, suggesting that strong biofilm production by certain non-streptococcal early colonizing taxa, including *L. saburreum***,** may reflect their adaptation to health-associated plaque rather than a direct caries-promoting phenotype. Accordingly, the observed pattern likely represents a species-specific ecological behavior rather than a contradiction of caries-associated biofilm concepts. These findings further support the hypothesis that carious lesions may indicate altered resistance properties of the supragingival biofilm. The significant results for *L. saburreum* can primarily be attributed to the fact that isolates from the caries group were predominantly categorized as non-biofilm formers.

In the previously mentioned study from our group, the relationship between antibiotic resistance and biofilm-forming capacity of oral bacteria was also investigated, employing partially different bacterial species and antibiotic agents (13). The relationship between biofilm-forming capacity and antimicrobial resistance was not consistent across species. This heterogeneity reflects the distinct ecological and metabolic characteristics of facultative and obligate anaerobes, which shape biofilm architecture and resistance behavior in species-specific ways. As a result, the observed associations between biofilm formation and resistance cannot be generalized across taxa and should be interpreted within the ecological context of each species. Differences in oxygen tolerance, metabolic specialization, and ecological niche adaptation can shape both biofilm phenotype and resistance behavior independently of cariogenic activity. Furthermore, potential confounders including age distribution and the unequal species composition between the groups may have contributed to these associations. These factors should be considered when interpreting isolate-level relationships between biofilm formation and antibiotic resistance. Given the considerable variability observed across species and antibiotic agents, broad conclusions regarding the relationship between caries status, antimicrobial resistance, and biofilm formation are limited. These associations appear to be species-specific and should therefore be interpreted within the ecological and metabolic context of each taxon rather than generalized across the supragingival microbiota. Overall, the results only partially support the original hypothesis that caries status would be associated with higher antimicrobial resistance and stronger biofilm formation across the supragingival microbiota. The observed associations were predominantly species-specific rather than universal, indicating that caries-related ecological changes do not uniformly translate into resistance or biofilm phenotypes across all taxa.

In above mentioned study, aerobic species exhibited stronger biofilm formation than anaerobic species, which is consistent with the findings of the present study. Al-Ahmad et al. further demonstrated that most isolates with elevated MIC values were classified as moderate or strong biofilm formers. In the present study, such an association could not be observed for facultative anaerobic species. However, among obligate anaerobic bacteria, a higher proportion of resistant outcomes was likewise associated with moderate and strong biofilm formers. This relationship may be explained by enhanced horizontal gene transfer within biofilms (11, 13, 33).

Further research is required to elucidate the impact of the altered antibiotic resistance and biofilm-forming capacity of *V. parvula* and *L. saburreum* in caries patients on the resistance of the supragingival biofilm as a whole.

## Conclusion

In the present study, both antimicrobial susceptibility testing and biofilm formation assays revealed differences between the caries and healthy groups for *V. parvula* and *L. saburreum*. Moreover, the findings indicate an association between biofilm formation and antibiotic resistance in obligate anaerobic bacterial species. Strong and moderate biofilm formers exhibited a higher proportion of resistant outcomes compared to non-biofilm formers. This appears to be a consequence of increased horizontal gene transfer and supports the observation that bacteria within a biofilm are more resistant to antimicrobial agents. Assuming that the manifestation of caries requires the persistence of a supragingival biofilm over an extended period, caries may serve as an indicator of altered resistance patterns in oral bacteria. Whether the observed differences in resistance behavior and biofilm-forming capacity contribute to increased resistance of the supragingival biofilm as a whole could not be demonstrated in this study, as the complex composition of the oral biofilm can only be partially reproduced using *in vitro* methods. Further research is needed to clarify the influence of the altered antibiotic resistance and biofilm-forming capacity of *V. parvula* and *L. saburreum* in caries patients on the microbial composition of supragingival biofilms. Future studies should consider including a higher number of isolates cultivated from a greater number of participants.

## Data Availability

The raw data supporting the conclusions of this article will be made available by the authors, without undue reservation.

## Supplementary material

Supplementary Table S1 Biofilm forming capacity of *V. parvula* Isolates. H = healthy group; C = caries group; Well 1 - 4 = replicate OD measurements; Mean Blank=mean blank OD value; SD Blank=standard deviation of the blank OD value; Low cut-off / High cut-off=thresholds for biofilm classification.

Supplementary Table S2 Biofilm forming capacity of *L. saburreum* Isolates. H = healthy group; C = caries group; Well 1 - 4 = replicate OD measurements; Mean Blank=mean blank OD value; SD Blank, standard deviation of the blank OD value; Low cut-off / High cut-off=thresholds for biofilm classification.
