# Supplementary material for: Antibiotic resistance and biofilm forming capacity of supragingival bacteria in healthy and caries patients
Source: Front Oral Health. 2026 Apr 24;7:1800312. doi: 10.3389/froh.2026.1800312 (PMC13154273; doi:10.3389/froh.2026.1800312)
Supplement: Supplementary Table S1 — Biofilm forming capacity of V. parvula Isolates. H = healthy group; C = caries group; Well 1 - 4 = replicate OD measurements; Mean Blank=mean blank OD value; SD Blank=standard deviation of the blank OD value; Low cut-off / High cut-off=thresholds for biofilm classification. [file Table1.docx]

| Group | Isolate ID | Well 1 | Well 2 | Well 3 | Well 4 | Mean Blank | SD Blank | Low cut-off | High cut-off | Biofilm category |
| --- | --- | --- | --- | --- | --- | --- | --- | --- | --- | --- |
| H | HG-03-5ba | 0.404100001 | 0.382600009 | 0.361999989 | 0.342900008 | 0.149025001 | 0.105563861 | 0.465716584 | 1.397149752 | C1 |
| H | FG-11-6a | 0.209199995 | 0.469099998 | 0.217500001 | 0.183599994 | 0.149025001 | 0.105563861 | 0.465716584 | 1.397149752 | C1 |
| H | HG-08-6a | 0.480599999 | 0.513999999 | 1.069200039 | 0.454699993 | 0.149025001 | 0.105563861 | 0.465716584 | 1.397149752 | C2 |
| H | FG-01-AP-P-6a | 0.550100029 | 0.721599996 | 0.689700007 | 0.851999998 | 0.149025001 | 0.105563861 | 0.465716584 | 1.397149752 | C2 |
| H | FG-02-MH-BC-F-6a | 0.186800003 | 0.217199996 | 0.230800003 | 0.183699995 | 0.149025001 | 0.105563861 | 0.465716584 | 1.397149752 | C1 |
| H | FG-03-SP-6a | 0.290100008 | 0.330900013 | 0.330900013 | 0.400599986 | 0.149025001 | 0.105563861 | 0.465716584 | 1.397149752 | C1 |
| H | HG-02-MA-6a | 0.406699985 | 0.365300000 | 0.340799987 | 0.385500014 | 0.166175000 | 0.087337866 | 0.428188597 | 1.284565792 | C1 |
| H | HG-04-SS-6a | 0.411000013 | 0.386099994 | 0.550000012 | 0.385800004 | 0.166175000 | 0.087337866 | 0.428188597 | 1.284565792 | C2 |
| H | FG-04-HB-6a | 0.367599994 | 0.339100003 | 0.314000010 | 0.283499986 | 0.166175000 | 0.087337866 | 0.428188597 | 1.284565792 | C1 |
| H | FG-05-6a | 0.345699996 | 0.344700009 | 0.287499994 | 0.373899996 | 0.166175000 | 0.087337866 | 0.428188597 | 1.284565792 | C1 |
| H | FG-06-6a | 0.431100011 | 0.381000012 | 0.379599988 | 0.396699995 | 0.166175000 | 0.087337866 | 0.428188597 | 1.284565792 | C1 |
| H | FG-07-6a | 0.321200013 | 0.288599998 | 0.265100002 | 0.286599994 | 0.166175000 | 0.087337866 | 0.428188597 | 1.284565792 | C1 |
| H | FG-07-V16-6aa | 0.380299985 | 0.509700000 | 0.470999986 | 0.316700011 | 0.166175000 | 0.087337866 | 0.428188597 | 1.284565792 | C1 |
| H | FG-05-V16-5ba | 0.321000010 | 0.303200006 | 0.311899990 | 0.387400001 | 0.166175000 | 0.087337866 | 0.428188597 | 1.284565792 | C1 |
| H | HG-10-6aa | 0.365700006 | 0.278200001 | 0.376199991 | 0.293099999 | 0.166175000 | 0.087337866 | 0.428188597 | 1.284565792 | C1 |
| H | HG-12-6a | 0.336400002 | 0.418900013 | 0.393099993 | 0.421700001 | 0.166175000 | 0.087337866 | 0.428188597 | 1.284565792 | C1 |
| H | FG-13-6aa | 0.333400011 | 0.383399993 | 0.522300005 | 0.344399989 | 0.166175000 | 0.087337866 | 0.428188597 | 1.284565792 | C1 |
| H | FG-14-6a | 0.357300013 | 0.383500010 | 0.404700011 | 0.393099993 | 0.166175000 | 0.087337866 | 0.428188597 | 1.284565792 | C1 |
| H | FG-15-6a | 0.395599991 | 0.450300008 | 0.417499989 | 0.426699996 | 0.166175000 | 0.087337866 | 0.428188597 | 1.284565792 | C1 |
| H | FG-16-6a | 0.278699994 | 0.304399997 | 0.298099995 | 0.429699987 | 0.166175000 | 0.087337866 | 0.428188597 | 1.284565792 | C1 |
| H | FG-17-6a | 0.485199988 | 0.562200010 | 0.485399991 | 0.498199999 | 0.166175000 | 0.087337866 | 0.428188597 | 1.284565792 | C2 |
| H | FG-18-6a | 0.219999999 | 0.236000001 | 0.194800004 | 0.202099994 | 0.166175000 | 0.087337866 | 0.428188597 | 1.284565792 | C1 |
| C | FK-01-6a | 0.271499991 | 0.282099992 | 0.311300009 | 0.305799991 | 0.153450001 | 0.014084624 | 0.195703873 | 0.587111618 | C2 |
| C | FK-02-6a | 0.312900007 | 0.308600008 | 0.313800007 | 0.331600010 | 0.153450001 | 0.014084624 | 0.195703873 | 0.587111618 | C2 |
| C | FK-04-6a | 0.238499999 | 0.211899996 | 0.248699993 | 0.255299985 | 0.153450001 | 0.014084624 | 0.195703873 | 0.587111618 | C2 |
| C | FK-05-5a | 0.228799999 | 0.233099997 | 0.227200001 | 0.218199998 | 0.153450001 | 0.014084624 | 0.195703873 | 0.587111618 | C2 |
| C | FK-06-6aa | 0.212599993 | 0.236399993 | 0.236399993 | 0.236399993 | 0.153450001 | 0.014084624 | 0.195703873 | 0.587111618 | C2 |
| C | FK-07-5ba | 0.241099998 | 0.234899998 | 0.224600002 | 0.266499996 | 0.153450001 | 0.014084624 | 0.195703873 | 0.587111618 | C2 |
| C | HK-03-6a | 0.283899993 | 0.318199992 | 0.280800015 | 0.344999999 | 0.153450001 | 0.014084624 | 0.195703873 | 0.587111618 | C2 |
| C | HK-06-6aa | 0.139200002 | 0.109899998 | 0.115800001 | 0.145199999 | 0.153450001 | 0.014084624 | 0.195703873 | 0.587111618 | C1 |
| C | HK-08-6a | 0.133499995 | 0.152600005 | 0.152700007 | 0.186499998 | 0.153450001 | 0.014084624 | 0.195703873 | 0.587111618 | C1 |
| C | HK-01-6aa | 0.285200000 | 0.239099994 | 0.254200011 | 0.242400005 | 0.153450001 | 0.014084624 | 0.195703873 | 0.587111618 | C2 |
| C | HK-02-6aa | 0.226300001 | 0.241799995 | 0.282599986 | 0.283300012 | 0.153450001 | 0.014084624 | 0.195703873 | 0.587111618 | C2 |
| C | HK-05-6ba | 0.371100008 | 0.392300010 | 0.425399989 | 0.429100007 | 0.127699999 | 0.033848390 | 0.229245168 | 0.687735504 | C2 |
| C | HK-07-6a | 0.469500005 | 0.494899988 | 0.486000001 | 0.437099993 | 0.127699999 | 0.033848390 | 0.229245168 | 0.687735504 | C2 |
| C | HK-09-11a | 0.105300002 | 0.183799997 | 0.121799998 | 0.116200000 | 0.127699999 | 0.033848390 | 0.229245168 | 0.687735504 | C1 |
