# Supplementary material for: Antibiotic resistance and biofilm forming capacity of supragingival bacteria in healthy and caries patients
Source: Front Oral Health. 2026 Apr 24;7:1800312. doi: 10.3389/froh.2026.1800312 (PMC13154273; doi:10.3389/froh.2026.1800312)
Supplement: Supplementary Table S2 — Biofilm forming capacity of L. saburreum Isolates. H = healthy group; C = caries group; Well 1 - 4 = replicate OD measurements; Mean Blank=mean blank OD value; SD Blank, standard deviation of the blank OD value; Low cut-off / High cut-off=thresholds for biofilm classification. [file Table2.docx]

| Group | Isolate ID | Well 1 | Well 2 | Well 3 | Well 4 | Mean Blank | SD Blank | Low cut-off | High cut-off | Biofilm category |  |
| --- | --- | --- | --- | --- | --- | --- | --- | --- | --- | --- | --- |
| H | FG-11-10a | 0.215499997 | 0.444299996 | 0.146300003 | 0.540899992 | 0.169300005 | 0.069410182 | 0.377530551 | 1.132591654 | C1 |  |
| H | HG-08-10a | 0.181600004 | 0.863600016 | 0.779100001 | 0.377999991 | 0.169300005 | 0.069410182 | 0.377530551 | 1.132591654 | C2 |  |
| H | FG-12-12a | 0.229399994 | 0.121399999 | 0.119900003 | 0.109099999 | 0.127699999 | 0.033848390 | 0.229245168 | 0.687735504 | C1 |  |
| H | FG-01-AP-P-C15a | 0.221699998 | 1.277300000 | 0.174500003 | 0.421200007 | 0.169300005 | 0.069410182 | 0.377530551 | 1.132591654 | C2 |  |
| H | FG-02-MH-BC-F-C10a | 0.697899997 | 0.197999999 | 0.305000007 | 0.798099995 | 0.169300005 | 0.069410182 | 0.377530551 | 1.132591654 | C2 |  |
| H | FG-04-HB-C14a | 0.591700017 | 0.728699982 | 0.347400010 | 0.144899994 | 0.169300005 | 0.069410182 | 0.377530551 | 1.132591654 | C2 |  |
| H | FG-06-14a | 0.179499999 | 0.204999998 | 0.559499979 | 0.560199976 | 0.169300005 | 0.069410182 | 0.377530551 | 1.132591654 | C1 |  |
| H | FG-07-13a | 0.131899998 | 0.136299998 | 0.145099998 | 0.127100006 | 0.169300005 | 0.069410182 | 0.377530551 | 1.132591654 | C1 |  |
| H | HG-12-11a | 4.000000000 | 3.986799955 | 3.823199987 | 3.742500067 | 0.166175000 | 0.087337866 | 0.428188597 | 1.284565792 | C3 |  |
| H | FG-14-12a | 3.362499952 | 2.794100046 | 2.183599949 | 2.546999931 | 0.166175000 | 0.087337866 | 0.428188597 | 1.284565792 | C3 |  |
| H | FG-13-10a | 2.386100054 | 1.683099985 | 1.427899957 | 1.689700007 | 0.099250000 | 0.007441103 | 0.121573308 | 0.364719923 | C3 |  |
| H | FG-16-9a | 0.697700024 | 0.459199995 | 0.851100028 | 0.674399972 | 0.099250000 | 0.007441103 | 0.121573308 | 0.364719923 | C3 | |
| H | FG-17-9a | 0.154400006 | 0.131699994 | 0.144600004 | 0.119999997 | 0.099250000 | 0.007441103 | 0.121573308 | 0.364719923 | C2 |  |
| H | FG-18-11a | 2.945899963 | 4.000000000 | 3.902699947 | 4.000000000 | 0.099250000 | 0.007441103 | 0.121573308 | 0.364719923 | C3 |  |
| C | FK-01-10a | 0.152199998 | 0.262199998 | 0.160899997 | 0.159199998 | 0.179825000 | 0.091651530 | 0.454779589 | 1.364338768 | C1 |  |
| C | FK-02-10a | 0.394499987 | 0.607400000 | 0.293399990 | 0.370999992 | 0.179825000 | 0.091651530 | 0.454779589 | 1.364338768 | C1 |  |
| C | FK-04-8a | 0.129199997 | 0.110699996 | 0.221799999 | 0.106700003 | 0.179825000 | 0.091651530 | 0.454779589 | 1.364338768 | C1 |  |
| C | FK-05-10a | 0.188999996 | 0.342599988 | 0.223800004 | 0.521300018 | 0.179825000 | 0.091651530 | 0.454779589 | 1.364338768 | C1 |  |
| C | FK-06-8a | 0.306300014 | 0.238700002 | 0.892300010 | 0.576799989 | 0.179825000 | 0.091651530 | 0.454779589 | 1.364338768 | C2 |  |
| C | FK-08-12a | 0.134800002 | 0.139300004 | 0.108499996 | 0.884199977 | 0.179825000 | 0.091651530 | 0.454779589 | 1.364338768 | C1 |  |
| C | HK-03-8a | 0.271800011 | 0.329100013 | 0.236699998 | 0.114200003 | 0.179825000 | 0.091651530 | 0.454779589 | 1.364338768 | C1 |  |
| C | HK-08-13a | 0.114000000 | 0.108599998 | 0.117399998 | 0.171599999 | 0.127699999 | 0.033848390 | 0.229245168 | 0.687735504 | C1 |  |
| C | HK-01-12a | 0.110100001 | 0.113600001 | 0.106100000 | 0.110600002 | 0.127699999 | 0.033848390 | 0.229245168 | 0.687735504 | C1 |  |
| C | HK-06-12a | 0.662800014 | 0.291799992 | 0.308299989 | 0.302899987 | 0.149025001 | 0.105563861 | 0.465716584 | 1.397149752 | C1 |  |
| C | HK-07-14a | 0.320899993 | 0.328799993 | 0.268200010 | 0.236100003 | 0.149025001 | 0.105563861 | 0.465716584 | 1.397149752 | C1 |  |
| C | HK-09-9a | 0.456600010 | 0.093699999 | 0.274899989 | 0.287800014 | 0.149025001 | 0.105563861 | 0.465716584 | 1.397149752 | C1 |  |
